# Predicting risk factors of non-utilisation of postnatal care in three neighbouring East African countries: application of the decision tree

**DOI:** 10.1136/bmjph-2024-001497

**Published:** 2025-10-15

**Authors:** Chenai Mlandu, Zvifadzo Matsena-Zingoni, Eustasius Musenge

**Affiliations:** 1 School of Public Health, University of the Witwatersrand, Faculty of Health Sciences, Johannesburg, South Africa; 2 Center for Biomedical Modeling, Department of Psychiatry and Biobehavioral Sciences, University of California Los Angeles David Geffen School of Medicine, Los Angeles, California, USA

**Keywords:** Community Health, Health Services Accessibility, Cross-Sectional Studies

## Abstract

**Introduction:**

Postnatal care (PNC) is recommended for the optimal health of mothers and newborns; however, PNC uptake remains poor in sub-Saharan Africa. Traditional statistical approaches have been used to predict healthcare service utilisation more often but are limited in examining complex relationships compared with decision tree (DT) models.

**Objectives:**

This study aims to predict the main risk factors of PNC non-utilisation in three neighbouring East African countries using the DT models.

**Methods:**

PNC non-utilisation meant that both the mother and neonate did not receive at least one postnatal check within 6 weeks after delivery. Demographic and Health Surveys data from the Democratic Republic of Congo (DRC) (2013/2014), Kenya (2014) and Tanzania (2015/2016) were used. The DT model’s predictive performance was compared with the standard logistic regression (LR) using accuracy, sensitivity, specificity and area under the receiver operating characteristic curve.

**Results:**

DT models exhibited higher accuracy and sensitivity than LR models. In the DRC, women with low-quality antenatal care (ANC), home deliveries and unemployment had the highest probability of PNC non-utilisation (92.0%). In Kenya, women who had home deliveries, unemployment and limited access to mass media showed the highest likelihood of PNC non-utilisation (87.0%). In Tanzania, women with home deliveries, low-quality ANC and unwanted pregnancies exhibited the highest likelihood of PNC non-utilisation (100.0%).

**Conclusion:**

Women with low-quality ANC, home deliveries, unemployment, unwanted pregnancies and limited access to mass media were classified as high-risk groups of PNC non-utilisation. These findings can help prioritise interventions and enhance PNC uptake in East Africa. Additionally, DT models can be applied as valuable tools for predicting maternal and child healthcare services utilisation in other sub-Saharan African countries.

**WHAT IS ALREADY KNOWN ON THIS TOPIC**

**WHAT THIS STUDY ADDS**

- Although there are studies on factors associated with low uptake of PNC, these studies have often used regression methods with limited capacity to analyse complex relationships or interactions. In contrast, decision tree (DT) models are ideally suited for examining complex interactions among factors, and their application can aid in classifying and prioritising women at high risk of not using PNC for more effective targeted interventions.

**HOW THIS STUDY MIGHT AFFECT RESEARCH, PRACTICE OR POLICY**

- Using the DT models, the study identified high-risk populations of non-utilisation of PNC, such as women with poor quality antenatal care, home deliveries, unemployment, unwanted pregnancies and no access to mass media. These findings can be used to prioritise interventions in high-risk populations in East Africa, thereby optimising public health resources and increasing PNC uptake.

## Introduction

 The rates of child mortality are high in the initial month following birth.^1^ Globally, about 2.5 million children died in the first month of life in 2017, approximately 7000 every day.^1^ Sub-Saharan Africa (SSA) experiences the highest number of newborn deaths. The region recorded the highest neonatal mortality rate (NMR) in 2017 at 27 deaths per 1000 live births, followed by Southern Asia with 26 deaths per 1000 live births.^1^ Also, NMR is unevenly spread across SSA, with Eastern African nations accounting for nearly half of all newborn deaths.^2^ The major causes of newborn deaths are preterm birth complications, intrapartum-related events and infections.^3^ Most newborn deaths result from preventable and treatable causes.^3^

The WHO defines the postnatal period as the 6 weeks following a child’s birth.^4^ This period is considered critical for both the mother and the child because majority of maternal and neonatal deaths occur during this period.^4 5^ Timely attendance of postnatal care (PNC) allows health practitioners to recognise and address potential health problems, such as neonatal infections like sepsis and pneumonia, feeding difficulties and developmental issues that increase the risks of morbidity and mortality in newborns.^4 6^ Furthermore, timely PNC provides an opportunity to get health information and support for beneficial practices, such as newborn care and exclusive breastfeeding, which are beneficial to the newborn’s health and growth.^4^ However, less than half of the mothers and newborns do not use PNC in SSA, including East Africa, and thereby forfeit these benefits.^7^

Studies conducted in SSA have found several factors to be significantly associated with non-utilisation of PNC, including older age, being unemployed, low maternal education, rural residency, long health facility travel distances, fewer antenatal care (ANC) visits, giving birth at home and complications during labour.^7 8^ Additionally, limited access to mass media, low decision-making power of women, traditional or cultural practices and religion are among the common driving factors.^7 9 10^

Although several studies have identified factors that contribute to non-utilisation of PNC,^7 9 10^ associations are often examined using traditional regression techniques, such as logistic regression (LR) models. These models are rarely used to evaluate complex interactions between factors, due to computational limitations and difficulty in interpretation. Data mining methods like the decision tree (DT) models offer unique advantages over regression techniques because of their ability to deal with non-linear data and analyse complex interactions based on the combinations of risk factors, which helps to identify high-risk populations for targeted intervention efforts.^11^ Notably, no recent studies have employed data mining methods like the DT models to predict the non-utilisation of PNC.^12^ The use of DT models will help to create the risk profiles of women at high risk of PNC non-utilisation for more effective targeted interventions. Therefore, this study aimed to predict the main risk factors of non-utilisation of PNC among reproductive-age women in the Democratic Republic of Congo (DRC), Kenya and Tanzania using a DT model.

## Methods and materials

### Study design and sampling procedure

The study used secondary data from cross-sectional Demographic and Health Survey (DHS) surveys from three neighbouring East African countries, namely the DRC (2013/2014), Kenya (2014) and Tanzania (2015/2016).^13 14^ The DHS is funded by the US Agency for International Development, which collects information on sociodemographic characteristics and health indicators, including maternal and child health.^15^ Although our analysis represents PNC utilisation patterns from 2013 to 2016, it is still relevant for guiding interventions in SSA because the DHS provides the most comparable and representative data in SSA. Also, key structural determinants of PNC utilisation like maternal education, rural–urban residence and household wealth remain key drivers of service uptake, making the finding relevant for guiding future interventions.

Two-stage cluster sampling was carried out in the DHS surveys. In the first stage, enumeration areas (EAs) or clusters were selected with probabilities equal to the EA or cluster size, and in the second stage, households were selected per cluster with equal-probability systematic.^16^ Women aged 15–49 years in selected households were eligible to be interviewed. A total of 8940, 6596 and 6873 women from the DRC, Kenya and Tanzania, respectively, were considered for analysis.

### Inclusion and exclusion criteria

The study comprised women aged 15–49 years who had a live birth in the past 5 years preceding DHS surveys in the DRC, Kenya and Tanzania. Only women who reported on ANC, skilled birth attendance (SBA) and PNC were included in the study. Only information related to the most recent birth was used.

### Study variables

#### Dependent variable

The dependent variable was non-utilisation of PNC. The mother and the neonate were said to have not used PNC if they did not receive at least one postnatal check within 6 weeks after delivery, used PNC if otherwise.^10^ It was constructed into a binary variable coded as one (1) if the mother and neonate did not use PNC and zero (0) if both the mother and the neonate used PNC.

#### Explanatory variables

The explanatory variables were chosen based on the literature in SSA^7 9 10 17 18^ and availability in DHS surveys. The explanatory variables included place of residence (urban and rural), maternal age (15–24 years and 25–49 years), wanted pregnancy as a proxy for pregnancy intention (then, later and no more), maternal education (secondary and tertiary education, primary and no education), employment in the past year (yes and no), parity (one, two, three or more), exposure to mass media (yes and no), has a male partner (yes and no), head of household (male and female), household wealth (rich, middle and poor), household size (4 or less and 5 or more), financial decision-making (respondent, joint with husband/partner, husband/partner/other and has no partner), health insurance cover (yes and no), seeking medical help alone (no big problem and big problem), distance to the health facility (no big problem and big problem), medical costs (no big problem and big problem), timing of ANC (early if gestational age is less than 4 months and late if gestational age is 4 or more months), less than four ANC visits (no and yes), lacked SBA (no and yes) and place of delivery (facility and home). Quality of ANC was constructed using items on routine ANC services, including measurement of weight, height and blood pressure and collection of urine and blood samples. The items on routine ANC services were coded as binary responses (1=yes if the service was received and 0=no if otherwise). We then categorised women who scored>75% of the total score as having received low quality of ANC and coded as 1 and high quality of ANC if otherwise and coded as 0.

### Data management

The data were cleaned and edited using STATA/SE V.17.0. The data were weighted using the ‘svyset’ STATA command to account for the effect of the hierarchical nature of the DHS survey data and to restore the representativeness of the survey. A descriptive analysis of the variables was conducted. The data were then imported into R software to fit the DT model and LR. Stepwise regression was used to select independent variables that had a statistically significant relationship (p<0.05) with PNC non-utilisation. The selected significant independent variables were considered for the DT model and LR analysis.

The data were prepared for the machine learning (ML) analysis by splitting the data into training and testing datasets using an 80/20 split. The study outcome, PNC non-utilisation, was heavily imbalanced. ML methods perform well with balanced classes of the outcome. Thus, random oversampling examples were applied to the training datasets to correct the class imbalances.^19^ The random oversampling corrects class imbalance by randomly oversampling the minority class.^19^

The DT model was applied to the training data using the Recursive Partitioning and Regression Trees (rpart) package. The DT model selects the variables from the database to split the sample into progressively smaller subgroups, resulting in a multilevel structure that resembles a tree.^20^ When the DT model algorithm identifies the most important independent variable, the node divides into two branches until the next best variable is reached.^20^ A terminal node or leaf occurs when no remaining independent variable could yield a statistically significant difference (p<0.05) or no further split could be made due to the defined stopping rules. The DT stopping rules determine when the tree construction process should stop, preventing overfitting and limiting the tree size for simplicity. Common rules include pruning by using the complex parameter, limiting tree depth or minimum number of data points per node. In our study, the tree was pruned using the complex parameter from the cross-validation error curve, leaving only the most meaningful splits, that is, selected independent variables or features.^20^ For each of the nodes generated, the DT analysis computed the probabilities of the risk expressed as percentages. The LR analysis was conducted using the training data for comparison with the DT. The test data were used to validate the DT and LR models’ performance.

### Comparison between the LR and the DT model

LR and DT models are both used for classification problems in ML, but they differ in their approach and characteristics. LR is a linear model that estimates the probability of a binary outcome, while DTs create a hierarchical structure of rules to classify data. LR works well with linearly separable data, while DTs can capture non-linear relationships. LR has a limited ability to assess complex relationships due to computational and interpretational difficulties. However, DTs can examine complex interactions among factors based on the certain combinations of risk factors, which helps in understanding the complex interactions between factors and developing risk profiles for targeted interventions.^11^ Hence, DTs are gaining popularity within health research for decision-making, screening and diagnostics. They are also easy to interpret and visualise compared with other complex ML algorithms like support vector machines and artificial neural networks.^21^

The parameters used to compare the DT and LR models were accuracy, sensitivity, specificity and area under the receiver operating characteristic (ROC) curves. Accuracy is a metric that measures how often an ML model correctly predicts the outcome. Sensitivity is the proportion of true positive tests out of all individuals with the outcome. Specificity is the proportion of true negatives out of all individuals without the outcome.^22^ An ROC curve is a plot of the sensitivity versus 1−specificity of a test. An area under the ROC curve (AUC) is an effective way to summarise the overall diagnostic accuracy of the test. It takes the value from 0 to 1, with 1 representing a perfectly accurate test and 0 representing a completely inaccurate one.^23^ AUC values of 0.5 indicate no discrimination, while 0.6≥AUC>0.5 indicates poor discrimination, 0.7≥AUC>0.6 indicates acceptable discrimination, 0.8≥AUC>0.7 indicates excellent discrimination and AUC>0.9 indicates exceptional discrimination.^23^ The larger the AUC, the better the overall performance of the model.

### Patient and public involvement

Patients or the public were not involved in the design, conduct, reporting or dissemination plans of our research.

### Results

Table 1 presents the characteristics of study participants in the DRC (8940), Kenya (6596) and Tanzania (6873).

**Table 1:** Characteristics of study participants

	DRC N(8940) n(%)	Kenya N(6596) n(%)	Tanzania N(6873) n(%)
Place of residence			
Urban	3212 (35.7)	2602 (39.9)	2083 (30.3)
Rural	5788 (64.3)	3910 (60.1)	4798 (69.7)
Maternal age			
Young women (18–24 years)	2756 (30.6)	1901 (29.2)	2249 (32.7)
Older women (25–49 years)	6244 (69.4)	4612 (70.8)	4632 (67.3)
Maternal education			
Primary and below	5086 (56.6)	4109 (63.1)	5754 (83.6)
Secondary and above	3914 (43.5)	2402 (36.9)	1127 (16.4)
Employment in the past 12 months			
No	1824 (20.3)	1851 (28.4)	1111 (16.2)
Yes	7175 (79.7)	4659 (71.6)	5770 (83.8)
Birth order			
One	4188 (46.5)	4495 (69.0)	4380 (63.7)
Two	3842 (42.7)	1744 (26.8)	2176 (31.6)
Three or more	971 (10.8)	273 (4.2)	325 (4.7)
Wanted pregnancy			
Then	6028 (67.0)	3933 (60.4)	4558 (66.3)
Late	2425 (27.0)	1818 (27.9)	1983 (28.8)
No more	546 (6.0)	761 (11.7)	340 (4.9)
Has a male partner			
No	1379 (15.3)	1194 (18.3)	1354 (19.7)
Yes	7621 (84.7)	5320 (81.7)	5592 (80.3)
Household head			
Male	7174 (79.7)	4491 (69.0)	5592 (81.3)
Female	1826 (20.3)	2023 (31.0)	1289 (18.7)
Household wealth			
Poor	3533 (39.3)	2443 (37.5)	2837 (41.2)
Middle	1821 (20.2)	1218 (18.7)	1316 (19.1)
Rich	3646 (40.5)	2850 (43.8)	2727 (39.7)
Household size			
4 or fewer	2172 (24.1)	3714 (41.7)	1898 (27.6)
5 or more	6828 (75.9)	3800 (58.3)	4983 (72.4)
Exposure to media			
No	4874 (54.2)	914 (14.0)	1175 (17.1)
Yes	4126 (45.8)	5597 (86.0)	5705 (82.9)
Financial decisions			
Respondent alone	687 (7.7)	441 (6.9)	242 (3.5)
Joint with husband/partner	3692 (41.6)	2494 (38.9)	2971 (43.4)
Husband/partner/other	3199 (35.1)	2280 (35.6)	2278 (33.3)
Has no partner	1379 (15.6)	1194 (18.6)	1354 (19.8)
Seeking medical help alone			
No big problem	6028 (67.0)	6106 (93.8)	5921 (86.1)
Big problem	2967 (33.0)	403 (6.2)	960 (13.9)
Travel distance to health facility			
No big problem	5471 (60.8)	4892 (75.1)	3776 (54.9)
Big problem	3524 (39.2)	1619 (24.9)	3105 (45.1)
Medical costs			
No big problem	2667 (29.6)	3963 (60.9)	3283 (47.7)
Big problem	6330 (70.4)	2548 (39.1)	3598 (52.3)
Health insurance cover			
No	8632 (95.9)	5336 (81.9)	6351 (92.3)
Yes	368 (4.1)	1176 (18.1)	530 (7.7)
Timing of ANC			
Early	3557 (39.5)	2723 (41.8)	3470 (50.4)
Late	5443 (60.5)	3790 (58.2)	3410 (49.6)
Less than four ANC visits			
No	4765 (53.2)	3946 (60.8)	3565 (52.1)
Yes	4191 (46.8)	2545 (39.2)	3283 (47.9)
ANC quality			
Low	4278 (47.5)	3775 (58.0)	3251 (47.2)
High	4723 (52.5)	2738 (42.0)	3630 (52.8)
Lacked SBA			
No	967 (10.7)	1009 (15.5)	2316 (33.7)
Yes	8034 (89.3)	5502 (84.5)	4564 (66.3)
Place of delivery			
Facility	7920 (12.0)	4530 (69.6)	4742 (68.9)
Home	1080 (88.0)	1981 (30.4)	2139 (31.1)
Mode of delivery			
Normal	8424 (93.7)	5886 (90.5)	6401 (93.0)
Caesarean section	566 (6.3)	620 (9.5)	480 (7.0)
Non-utilisation of PNC			
No	1381 (15.4)	3007 (46.2)	5442 (20.9)
Yes	7619 (84.6)	3506 (53.8)	1439 (79.1)

ANC, antenatal care; DRC, Democratic Republic of Congo; PNC, postnatal care; SBA, skilled birth attendance.

### Decision tree

**Figure 1:**
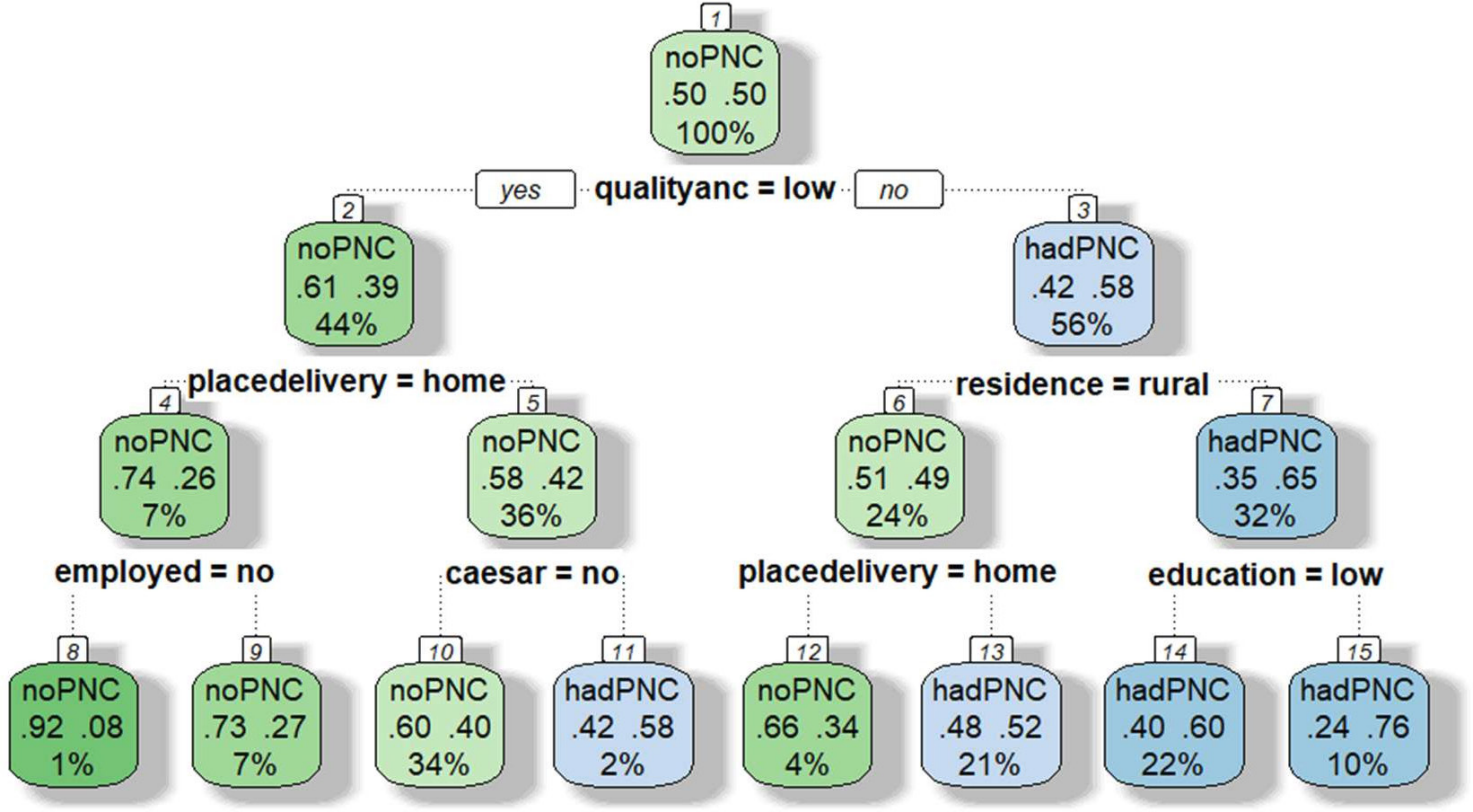
DT for predicting the risk of non-utilisation of PNC among women in the DRC. (1) Root (2) Low-quality ANC (3) High-quality ANC (4) Home delivery (5) Facility delivery (6) Rural residence (7) Urban residence (8) No employment (9) Had employment (10) Normal delivery (11) Caesarean section (12) Home delivery (13) Facility delivery (14) Primary or no education (15) Secondary or higher education. Root node (top): This is the first split based on the quality of ANC. Branches: each split represents a condition (eg, quality of ANC=low). Leaves (end nodes) show the predicted outcome. Each leaf box shows (1) predicted class (eg, no PNC and had PNC), (2) probabilities (proportion of cases in that node) and (3) per cent of all cases in that node. ANC, antenatal care; DRC, Democratic Republic of Congo; DT, decision tree; PNC, postnatal care.

The DT model analysis is displayed in figures1^3^. In the DRC, six significant independent variables were selected to define further branches and classify the probability of non-utilisation of PNC: quality of ANC, place of delivery, mode of delivery, maternal education, place of residence and employment status, see figure 1. The DT model diagram above illustrates that women who did not use PNC are split into eight classifications or groups by reading the results of the diagram using the top-down stopping rule, that is, beginning with the parent node, quality of ANC. The DT algorithm chose the quality of ANC as the first variable to split the root node, dividing the population into two groups: women with low-quality ANC (44% of the sample) and high-quality ANC (56% of the sample), which were then segmented into eight classes. The probability of not using PNC for the combination of factors considered in the DT analysis ranged from 24.0% to 92.0%. Women who had high-quality ANC, lived in the urban areas and had secondary or higher education showed the lowest risk of not using PNC (24.0%). In contrast, women who had low-quality ANC, delivered birth at home and were unemployed had the highest probability of not using PNC (92.0%).

**Figure 2:**
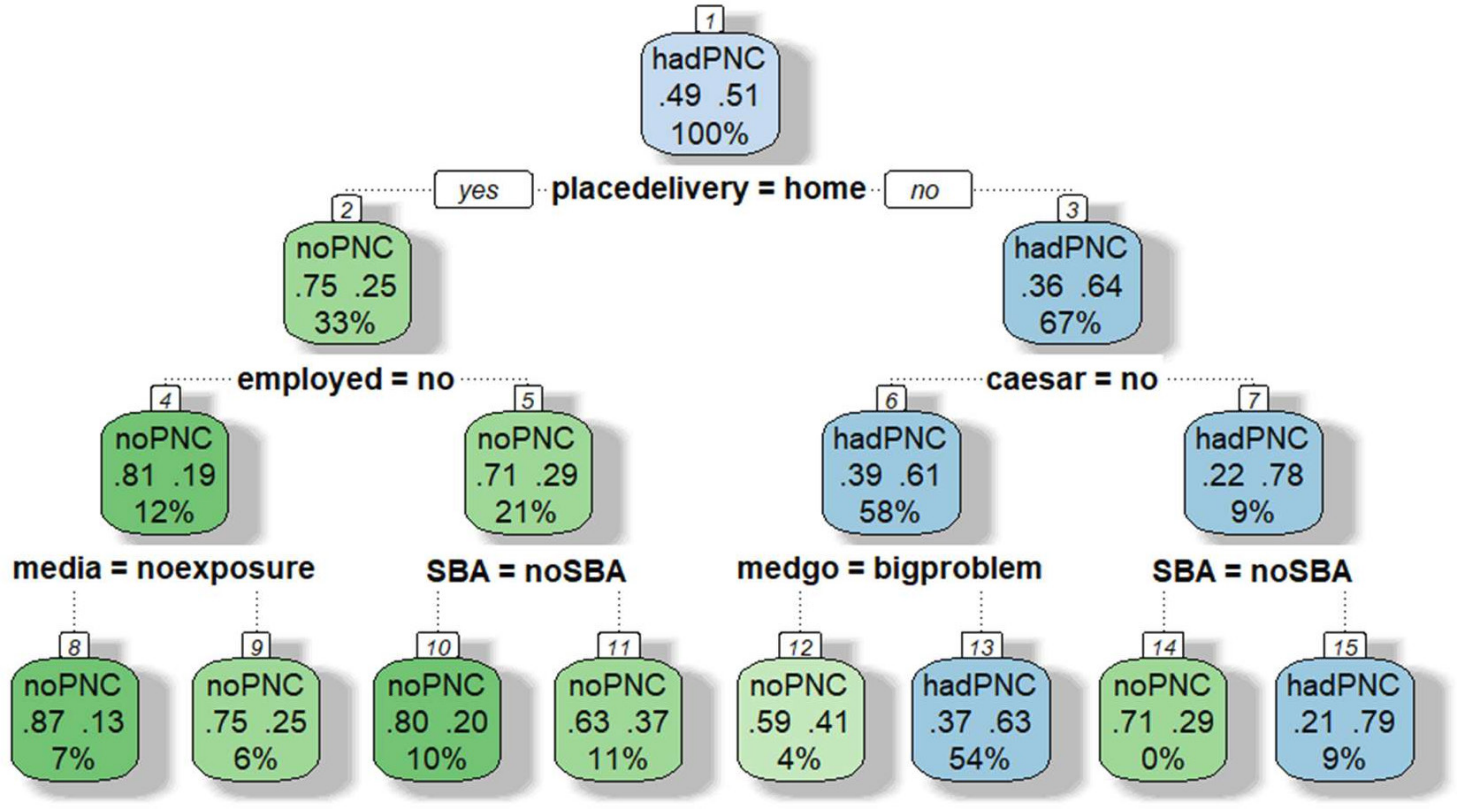
DT for predicting the risk of non-utilisation of PNC among women in Kenya. (1) Root (2) Home delivery (3) Facility delivery (4) No employment (5) Had employment (6) Normal delivery (7) Caesarean section (8) Non-exposure to mass media (9) Exposure to mass media (10) No SBA (11) Had SBA (12) Big problem seeking medical help alone (13) No big problem seeking medical help alone (14) No SBA (15) Had SBA . Root node (top): This is the first split based on the mode of delivery. Each split represents a condition (eg, place of delivery=home). Leaves (end nodes) show the predicted outcome. Each leaf box shows (1) predicted class (eg, no PNC and had PNC), (2) probabilities (proportion of cases in that node) and (3) per cent of all cases in that node. DT, decision tree; PNC, postnatal care; SBA, skilled birth attendance.

**Figure 3:**
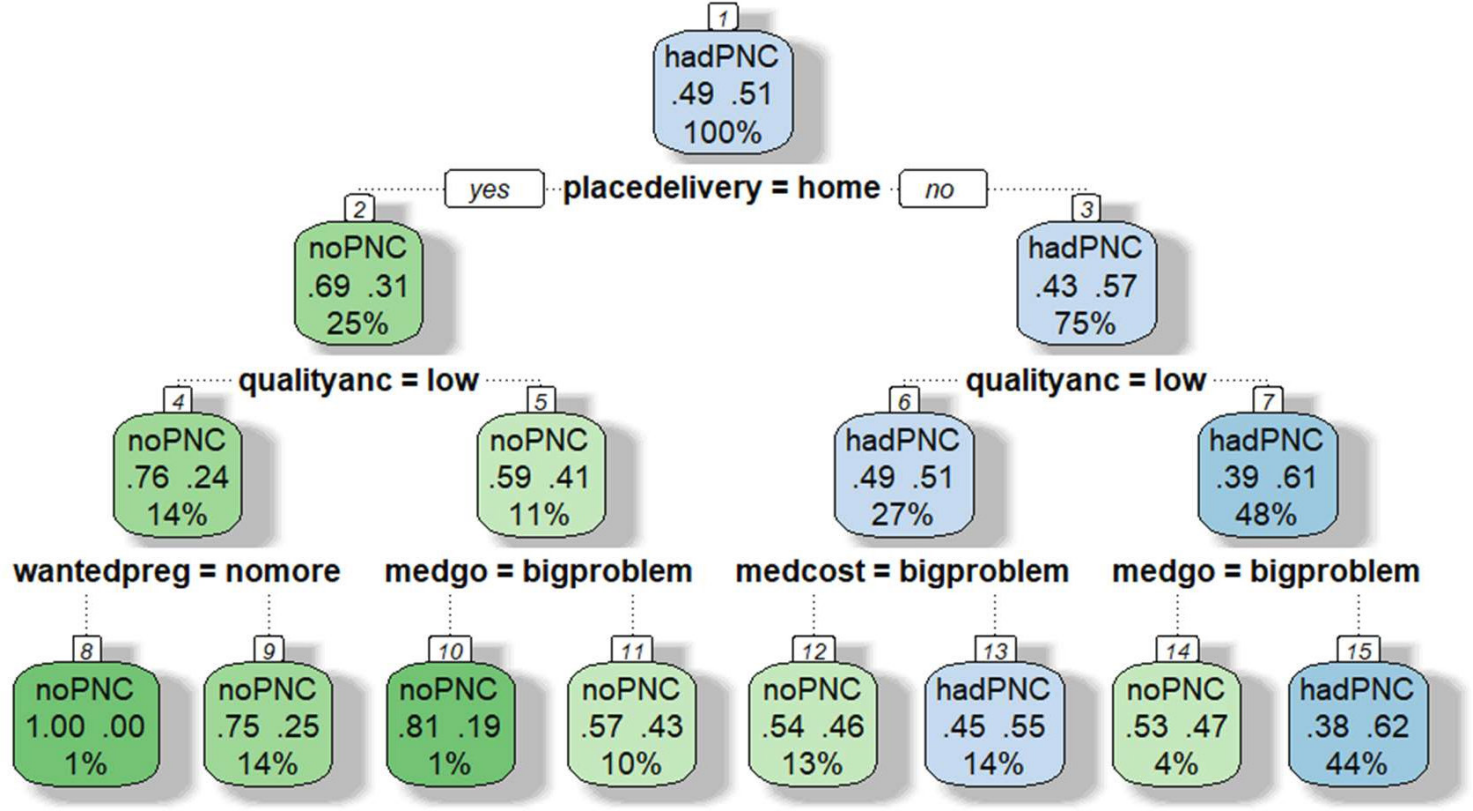
DT for predicting the risk of non-utilisation of PNC among women in Tanzania. (1) Root (2) Home delivery (3) Facility delivery (4) Low-quality ANC (5) High-quality ANC (6) Low-quality ANC (7) High-quality ANC (8) Wanted pregnancy no more (9) Wanted pregnancy then (10) Big problem with seeking medical help alone (11) No big problem with seeking medical help alone (12) Big problem with medical costs (13) No big problem with medical costs (14) Big problem with seeking medical help alone (15) No big problem with seeking medical help alone. Root node (top):This is the first split based on the mode of delivery. Each split represents a condition (eg, place of delivery=home). Leaves (end nodes) show the predicted outcome. Each leaf box shows (1) predicted class (eg, no PNC and had PNC), (2) probabilities (proportion of cases in that node) and (3) per cent of all cases in that node. ANC, antenatal care; DT, decision tree; PNC, postnatal care.

In Kenya, six significant independent variables were selected to define further branches and classify the probability of PNC non-utilisation: place of delivery, employment status, SBA, mode of delivery, media exposure and seeking medical help alone, see figure 2. The DT model chose the variable place of delivery as the first variable to split the root node, dividing the population into two groups: home delivery (33% of the sample) and facility delivery (66% of the sample), which were further separated into eight classes. The probability of not using PNC for the combination of factors considered in the DT analysis ranged from 21.0% to 87.0%. The classification with the lowest probability of non-utilisation of PNC included women who had facility deliveries, caesarean section and SBA (21.0%). However, women who delivered at home, were unemployed and had no exposure to mass media had the highest likelihood of not using PNC (87.0%).

In Tanzania, five significant independent variables were selected to define further branches and classify the probability of PNC non-utilisation: place of delivery, quality of ANC, seeking medical help alone, medical costs and wanted pregnancy, see figure 3. The DT chose the variable place of delivery as the first variable to split the root node, dividing the population into two groups: home delivery (25% of the sample) and facility delivery (75% of the sample), which were further separated into eight classes. The probability of not using PNC for the combination of factors considered in the DT analysis ranged from 38.0% to 100.0%. Women who delivered in facilities, received high-quality ANC and had no big problem with seeking medical help alone showed the lowest risk of not using PNC (38.0%). Women who delivered at home, received low-quality ANC and wanted pregnancy no more had the highest probability of not using PNC (100.0%).

### Performance of the DT and the LR models

The performance of DT and LR analysis for predicting PNC non-utilisation was evaluated using accuracy, sensitivity, specificity and AUC parameters, as shown in table 2. In the DRC, the DT model achieved an accuracy of 63.1%, sensitivity of 64.9% and specificity of 53.1%, while the LR obtained an accuracy of 57.8%, sensitivity of 58.0% and specificity of 57.0%. In Kenya, the DT model achieved an accuracy of 67.0%, sensitivity of 61.0% and specificity of 75.6%, while the LR obtained an accuracy of 66.7%, sensitivity of 57.2% and specificity of 79.6%. In Tanzania, the DT model achieved an accuracy of 61.4%, sensitivity of 60.3% and specificity of 65.8%, while the LR obtained an accuracy of 57.3%, sensitivity of 53.6% and specificity of 72.0%. The AUC values for the DT and LR models were 61.7% versus 60.2% in the DRC, 73.0% versus 74.8% in Kenya and 65.2% versus 65.6% in Tanzania. AUC values for both DT and LR models in the DRC, Kenya and Tanzania showed that the models could distinguish individuals who utilised or did not PNC (AUC>60%). Overall, the DT had better predictive perfomance in terms of accuracy and sensitivity than the LR in the DRC, Kenya and Tanzania.

**Table 2:** Performance parameters of the DT and LR models in the DRC, Kenya and Tanzania

	DRC		Kenya		Tanzania	
**Parameter**	LR	DT	LR	DT	LR	DT
Accuracy	58.9%	61.7%	66.7%	67.0%	57.3%	61.4%
Sensitivity	59.6%	64.9%	57.2%	61.0%	53.6%	60.3%
Specificity	55.1%	53.1%	79.6%	75.6%	72.0%	65.8%
AUC	60.2%	61.7%	74.8%	73.0%	65.6%	65.2%

AUC, area under the ROC curve; DRC, Democratic Republic of Congo; DT, decision tree; LR, logistic regression.

## Discussion

The study aimed to predict the main risk factors of non-utilisation of PNC among reproductive-age women using a DT model in the three neighbouring East African countries. Similar to previous research, the DT model had higher performance in accuracy and sensitivity compared with the LR model.^24^ The main advantage of DT consists of the ability to segment populations and examine complex interactions between multiple risk factors. In contrast, the LR emphasises the modelling of the probability of a binary outcome based on independent variables, often focusing on the overall predictive accuracy rather than detailed segmentation.^25^ Also, the DT model results are easy to visualise and interpret and often also expressed as a tree or decision rules.^20^ However, it is important to set a prior stopping rule to stop the DT model from growing into multiple levels, which could complicate its interpretation and may result in non-relevant splits.

In the DRC, the DT analysis showed that women with low-quality ANC, home deliveries and unemployment had the highest probability of non-utilisation of PNC (92.0%). It could be that women with low quality of care during pregnancy might be dissatisfied with the provision of care, resulting in them not seeking PNC. This could also be the reason why they would prefer giving birth at home. Barriers to access to care, like financial costs, could also hinder these women from utilising PNC, especially among unemployed women.^26^,^28^ These challenges in the DRC could be fuelled by a fractured health system, high medical costs and low job opportunities for women and high levels of unemployment.^2729^,^31^ To address these challenges, policy interventions should focus on strengthening the health system by developing and maintaining health facilities, particularly in underserved areas. Also, improving accessibility of care by removing user fees, expanding health insurance coverage and empowering women through education and economic opportunities can improve PNC uptake.

In Kenya, the DT analysis showed home deliveries, unemployment and non-exposure to mass media had the highest likelihood of PNC non-utilisation (87.0%). Possibly, women’s lack of PNC utilisation in Kenya is mainly driven by these factors because of the influence of harmful cultural norms, which favour home births over institutional deliveries and PNC services.^7 27^ Also, even with free access to maternal healthcare, transportation and distance to facilities, problems remain significant barriers for unemployed women in Kenya, which influence their decision to deliver at home and not seek PNC.^28^ Additionally, women in rural settings have limited exposure to mass media, which can hinder their awareness of PNC and its importance, leading to underutilisation of this service.^32^ These findings call for policy interventions aimed at improving institutional deliveries and addressing harmful cultural norms surrounding childbirth by engaging Community Health Workers to promote institutional deliveries and integrating home birth attendants (HBAs) into the formal health system. Additionally, financial incentives like conditional cash transfers or subsidies should be incorporated to support transportation costs and incentivise PNC visits. Also, PNC information can be incorporated into the existing health education programmes and community gatherings.

For Tanzania, the DT analysis showed that home deliveries, low-quality ANC and unwanted pregnancies exhibited the highest likelihood of PNC non-utilisation (100.0%). Challenges related to accessing maternal healthcare in Tanzania, particularly transportation costs and long distances to facilities, have been cited as major factors contributing to home deliveries leading to the underutilisation of PNC. These barriers also deter women from seeking PNC after childbirth.^7 33^ Also, health sector resource constraints like staff shortages and poor infrastructure at health facilities, particularly in rural settings, hinder its ability to enhance the quality of ANC, which deters women from seeking PNC.^34^ In addition, women with delayed or unwanted pregnancies often realise that they are pregnant late in pregnancy, which can result in delays in seeking ANC and have a negative impact on the subsequent utilisation of services, such as PNC.^35^ Our findings suggest the need for policies that enhance institutional deliveries and addressing barriers like distance, cost and cultural preferences for home births, particularly in rural settings. This could be done by improving infrastructure, providing transportation assistance and conducting community awareness programmes. Additionally, policies should also focus on enhancing the quality of ANC services by equipping health facilities with skilled healthcare workers and essential equipment. Furthermore, addressing delayed or unwanted pregnancies by expanding access to affordable family planning services, promoting reproductive health education as well as integrating maternal healthcare services with family planning services is crucial.

### Strengths and limitations

This study’s strength is its use of nationally representative data from multiple countries, enabling cross-country comparison and wider applicability. Additionally, the application of the DT can help identify and create risk profiles of women at high risk of not using PNC for effective targeted interventions. However, despite these strengths, this study has limitations. The main outcome on non-utilisation of PNC relies on women’s self-reports in the last 5 years before the survey and, thus, is subject to recall bias. The bias can be minimised by shortening the recall period and verifying information using maternal clinical records. The study did not evaluate other factors (features) of PNC non-utilisation that could potentially improve models’ performance, including health service factors, such as the availability of health facilities and skilled care workers and quality of obstetric and PNC care due to unavailability of data in DHS. Future studies should consider incorporating these variables as key factors of interest by linking community data with health service data. Although the DTs’ AUC values showed the models’ discriminative capacity, there is still room for improvement in future studies by incorporating potential key features or factors, such as health service factors that could improve the model’s ability to distinguish between classes. It is also challenging to establish temporal causality because the data are cross sectional.

## Conclusion

The DT model is an effective analytical method to complement the traditional LR. The DT model easily identified high-risk groups of women who could benefit from PNC interventions and improve uptake of PNC. Using the DT, in the DRC, women with low quality of ANC, home delivery and unemployment showed the highest probability of using PNC. In Kenya, women who delivered at home, were unemployed and had limited access to mass media showed the highest probability of not using PNC. For Tanzania, women with home deliveries, low quality of ANC and unwanted pregnancies showed the greatest probability of not using PNC. Government policymakers and stakeholders should consider targeted interventions, such as improving access and quality of care provided by health facilities, incorporating HBAs into the formal health system, creating more job opportunities for women and integrating maternal healthcare services with family planning services to improve PNC utilisation. Furthermore, DT models can be used as valuable tools for predicting maternal and child health services utilisation in other countries in SSA.

## Data Availability

Data are available in a public, open access repository.
